# High-Throughput Sequencing Methods for the Detection of Two Strawberry Viruses in Post-Entry Quarantine

**DOI:** 10.3390/v16101550

**Published:** 2024-09-30

**Authors:** Luciano Nunes-Leite, Lia W. Liefting, David W. Waite, Subuhi Khan, Jeremy R. Thompson

**Affiliations:** Plant Health and Environment Laboratory, Ministry for Primary Industries, P.O. Box 2095, Auckland 1140, New Zealand; david.waite@mpi.govt.nz (D.W.W.); subuhi.khan@mpi.govt.nz (S.K.)

**Keywords:** SMoV, SVBV, high-throughput sequencing, metatranscriptomics, tiled amplicon sequencing, PCR, rRNA depletion, plant quarantine

## Abstract

High-throughput sequencing (HTS) technologies may be a useful tool for testing imported plant germplasm for multiple pathogens present in a sample, offering strain-generic detection not offered by most PCR-based assays. Metatranscriptomics (RNAseq) and tiled amplicon PCR (TA-PCR) were tested as HTS-based techniques to detect viruses present in low titres. Strawberry mottle virus (SMoV), an RNA virus, and strawberry vein banding virus (SVBV), a DNA virus, were selected for comparison of RNAseq and TA-PCR with quantitative PCR assays. RNAseq of plant ribosomal RNA-depleted samples of low viral titre was used to obtain datasets from 3 M to 120 M paired-end (PE) reads. RNAseq demonstrated PCR-like sensitivity, able to detect as few as 10 viral copies/µL when 60 million (M) PE reads were generated. The custom TA-PCR primer panels designed for each virus were successfully used to recover most of the reference genomes for each virus. Single- and multiple-target TA-PCR allowed the detection of viruses in samples with around 10 viral copies/µL with a minimum continuous sequence length recovery of 500 bp. The limit of detection of the HTS-based protocols described here is comparable to that of quantitative PCR assays. This work lays the groundwork for an increased flexibility in HTS detection of plant viruses.

## 1. Introduction

The principle of exclusion of unwanted plant pathogens, to prevent the occurrence of plant diseases, has been the main guide to the establishment of plant quarantine services all over the world [1]. Post-entry quarantine (PEQ) is based on giving the perfect conditions to the assessed plant material for its growth and development, which are also the best conditions for the associated pathogens to induce symptoms of unwanted diseases [2]. Exclusion offered by PEQ facilities is one of the most valuable tools for protecting countries from unwanted plant diseases of plant material imported for various reasons, usually to become part of plant breeding programmes, to be used as crops, or to become part of public or private collections. [3,4]. Countries like Chile, Australia, and New Zealand are heavily invested in this approach to prevent the arrival of unwanted organisms with a potentially high impact on the primary sector [2,5,6,7,8,9].

Because of often cryptic symptoms and perennity in their host, viruses and virus-like pathogens are particularly important in PEQ screening worldwide, requiring extended periods during which plants are subjected to PEQ screening. The current screening for pathogens on imported germplasm is primarily based on polymerase chain reaction (PCR) methodologies [10]. PCR requires a priori knowledge of the pathogen’s genetic material and relies on exploiting relatively short conserved genomic regions for detection. The reliability, versatility, sensitivity, and specificity of PCR-based diagnostics have consolidated these assays as gold standards in the diagnostics of viruses worldwide [2,11].

The Plant Health and Environment Laboratory (PHEL) of the Ministry for Primary Industries (MPI) has been using the HTS Oxford Nanopore Technology (ONT) as an additional method to PCR for detection and confirmation of plant pathogens in symptomatic PEQ and domestic surveillance samples [12]. The clear benefits of ONT have been shown for samples that have mixed infections, new pathogens associated to a host or even undescribed pathogens at PHEL and other laboratories worldwide [12,13,14].

High-throughput sequencing (HTS) offers the opportunity to test imported plant germplasm for any pathogen present with high sensitivity [2,15]. HTS approaches for detection can offer flexibility to the type of nucleic acid used as input, and they require only that the sequences of pathogens of interest are present in global databases [12,16]. Nevertheless, a major challenge regarding the use of HTS as a standardised method for detection of plant pathogens is the current absence of guidelines for the validation of laboratory procedures and bioinformatic analyses required for compliance and repeatability [17,18]. Attempts were made to create guidelines and recommendations considering the analytical sensitivity and specificity required for comparing current and proposed testing methods for clinical virology [19,20] and for the diagnostics of plant viruses [21,22,23].

The application of HTS for virus diagnostics from plant material is enhanced by techniques that enrich for viral sequences since most RNA molecules present in nucleic acid extracts belong to the host itself and therefore crowd out the minority viral sequences. The depletion of host ribosomal RNA (ribodepletion) is a widely used technique for reducing this host RNA, thereby decreasing the depth of sequencing required for detection of foreign RNAs in low concentrations [24,25].

Whole genome amplification by tiled amplicon PCR (TA-PCR) has been proposed as a quick, flexible, and inexpensive method to enrich sequences for virus genomes using low depth sequencing approaches, such as Illumina MiSeq and ONT platforms [26]. TA-PCR protocols aim to amplify the whole genome of viruses present in low titres by a series of primers evenly distributed along their genome, increasing the proportion of reads matching target organisms [27,28,29,30]. TA-PCR is prized as a reliable epidemiological tool to investigate disease spread and evolution of viral pathogens. Widely used on studies of human and animal viruses, only a few reports describe the application of TA-PCR for whole genome amplification of plant pathogenic viruses, including for the detection of cucumber green mottle virus (CGMMV) [31] and for analysing the diversity of tomato brown rugose fruit virus (ToBRFV) [32].

We present here a quantitative comparison of proposed protocols of HTS-based methods to quantitative RT-PCR for the detection of plant viruses in low titre in strawberry. The positive single-stranded RNA (+ssRNA) virus strawberry mottle virus (species *Sadwavirus fragariae*) (SMoV) and the double-stranded DNA (dsDNA) virus strawberry vein banding virus (species *Caulimovirus venafragariae*) (SVBV) were used as target models in this study. RNAseq datasets obtained from ribodepleted total RNA samples were analysed to assess the minimum sequencing depth necessary for detection of viruses to a level comparable to PCR. TA-PCR was designed for two viruses and a single assay was performed to detect both viruses from samples with low viral titre. The sensitivity of the tested PCR and HTS protocols were compared in terms of viral copy numbers.

## 2. Materials and Methods

### 2.1. Plant Material

Fresh healthy strawberry (*Fragaria* x *ananassa*) leaves from plants maintained in an insect-proof greenhouse were used as healthy background for dilution of positive control samples. Positive control samples were obtained from fresh strawberry leaves infected with a strain of SMoV present in New Zealand [33]. Imported freeze-dried strawberry leaves infected with an uncharacterised strain of SVBV, kept at 4 °C, were the source of positive control, as SVBV is not present in New Zealand.

### 2.2. Nucleic Acid Extraction and Quantification

Total nucleic acid (TNA) was extracted from freeze-dried or fresh leaf tissue of strawberry. Plant tissue was homogenised in CTAB extraction buffer [34] at a 1:60 *w*/*v* ratio for freeze-dried material and 1:12 *w*/*v* ratio for fresh material. Homogenates were incubated at 65 °C for 10 min and centrifuged at 8000× *g* for 2 min, and the supernatant was used for extraction using the InviMag Plant DNA Mini Kit (Invitek GmbH, Berlin, Germany) on a KingFisher mL workstation (Thermo Scientific, Waltham, MA, USA) according to the manufacturer’s instructions. TNA samples were aliquoted and stored at −80 °C.

TNA extracts were assessed for contaminants by spectrophotometry (NanoDrop, Thermo Scientific) and RNA concentration was measured using a Qubit RNA High Sensitivity kit (Thermo Scientific). RNA quality number (RQN) was assessed using a DNF-472 HS RNA (15 nt) Kit (Agilent Technologies, Ankeny, IO, USA) on a Fragment Analyzer instrument (Agilent Technologies) prior to subsequent experiments.

### 2.3. Absolute Quantification of Viral RNAs

Quantitative reverse transcription PCR (RT-qPCR) was used for the quantification of SMoV and SVBV (Supplementary Table S1). Each sample was run for an internal control to verify competency of TNA for PCR amplification [35]. Samples with quantification cycle (Cq) equal to or lower than 35 for the respective target were considered true positives. Healthy strawberry TNA extracts were confirmed negative for both target viruses prior to use in subsequent experiments. RT-qPCR was performed with qScript XLT One-Step RT-qPCR ToughMix (Quantabio, Beverly, MA, USA). Reverse transcription was performed at 50 °C for 15 min followed by an initial denaturation step at 95 °C for 2 min followed by 40 cycles of 95 °C for 10 s (denaturation) and 60 °C for 45 s (annealing and extension). A non-template control sample and a positive control sample were included in each assay.

To produce a source of synthetic RNA for sensitivity testing, conventional PCR primers were designed to amplify regions containing the region targeted by the qPCR assays used above: 22 reference genomes for the RNA2 of SMoV were used to design primers (Supplementary Table S3), and 20 reference genomes were used to design primers for SVBV (Supplementary Table S4). Forward primers were designed with a T7 promoter sequence required by the RNA synthesis kit (Supplementary Table S1). Single-step reverse transcription PCR Amplification was performed with 10 µL GoTaq Green Master Mix (Promega, Madison, WI, USA), 1 µL of 10 mg/mL Bovine Serum Albumin (BSA), 0.3 µL of Rnasin Plus Ribonuclease Inhibitor (Promega), 1 µL of 0.1 M DTT (Invitrogen, Carlsbad, CA, USA), 2 µL of 10 µM working solutions of forward and reverse primers, 0.3 µL of SuperScript III Reverse Transcriptase (Invitrogen), and 2 µL of TNA, and final reaction volume was adjusted with sterile deionised water to 20 µL. An initial reverse transcription step performed at 50 °C for 30 min, with initial denaturation at 94 °C for 5 min, followed by 40 cycles of 94 °C for 30 s, 55 °C for 30 s, and 72 °C for 30 s, and final extension step at 72 °C for 10 min. After amplification, sequences were confirmed by Sanger sequencing, and RNA was synthesised using HiScribe T7 Quick High Yield RNA Synthesis Kit (New England Biolabs, Notting Hill, VIC, Australia) using these amplicons as templates. RNA cleanup and DNA removal were performed using the Monarch RNA Cleanup Kit (New England Biolabs). Synthetic RNA samples were aliquoted and kept at −80 °C.

Synthetic RNA standards for absolute quantification were prepared as a 10-fold serial dilution with Cq values ranging from 20 to 40. The diluent consisted of healthy strawberry total NA extract with total RNA concentration of 20 ng/µL.

### 2.4. Preparation of Samples for Assessment of Limit of Detection

Samples used to determine the limit of detection (LoD) of HTS-based diagnostics of viruses were prepared as tenfold serial dilution sets of positive controls for SMoV and SVBV. TNA extracts from healthy strawberry samples were used as the diluent. Final samples with RQN > 5 were adjusted to a final RNA concentration of 20 ng/µL. A set of four samples from the dilution series was chosen to be used in all assays to compare the LoD of distinct diagnostic methods for each virus: two dilutions with Cq lower than 35, one dilution at the RT-qPCR LoD of Cq ~ 35, and one dilution with Cq higher than 35—henceforth called sensitivity panel samples.

### 2.5. Meta-Transcriptomics for Detection of Viruses

The sensitivity panel samples were again assessed for RQN using a DNF-472 HS RNA (15 nt) Kit (Agilent Technologies) on a Fragment Analyzer instrument to confirm that they met the sequencing provider specifications. Prior to shipping, DNA was removed using the RapidOut DNA Removal Kit (Thermo Scientific) and concentration checked using the Qubit RNA HS Kit. RNA was prepared for shipment into RNA shipping tubes (RNAstable or RNA Ambient tubes) and dried using a vacuum desiccator for 2–4 h at room temperature.

Samples were sent for sequencing at Azenta Life Sciences (Suzhou, China—https://www.azenta.com (accessed on 23 May 2024)) or Novogene (Singapore—https://www.novogene.com/us-en/ (accessed on 23 May 2024)). The sequencing provider was requested to perform ribodepletion (Ribo-Zero rRNA Removal Kit (Illumina, San Diego, CA, USA)), library preparation (NEBNext Ultra RNA Library Prep Kit for Illumina (New England Biolabs)), and sequencing. The sequencing service we requested consisted of 120 M paired-end (PE) reads per sample obtained by Illumina NovaSeq PE150. 

The raw data were initially assessed for quality using FastQC (version 0.11.9, https://github.com/s-andrews/FastQC (accessed on 23 May 2024)). The remaining adapters present in the data were removed using Trimmomatic (version 0.39, https://github.com/timflutre/trimmomatic (accessed on 23 May 2024)) and the filtered data were checked for quality again using FastQC. The filtered reads were then subsampled in triplicates into files of 3 M, 7 M, 15 M, 30 M, 40 M, and 60 M PE reads to be tested for the minimum number of reads required for consistent and reliable detection of the targeted organisms. Reads from each of these files, and from the original files with 120 M PE reads, were then mapped to reference genomes of each of the targeted viruses by BWA (version 0.7.17, https://github.com/lh3/bwa (accessed on 23 May 2024)) and data filtering and reporting was performed with SAMtools (version 1.15.1-GCC-11.3.0, https://github.com/samtools/samtools (accessed on 23 May 2024)). The identity of these reads was validated by de novo assembly of these mapped reads into contigs using SPAdes (version 3.15.4, https://github.com/ablab/spades (accessed on 23 May 2024)) and the assembled contigs were compared to the NCBI database by BLAST+ (version 2.13.0, https://ftp.ncbi.nlm.nih.gov/blast/executables/blast+/LATEST/ (accessed on 23 May 2024)). When assembly was not possible, the unassembled reads were identified by BLAST+. In both cases, TaxonKit (version 0.13.0, https://github.com/shenwei356/taxonkit (accessed on 23 May 2024)) was used to produce human readable tables of contigs or reads and their respective identities.

### 2.6. Development of Tiled-Amplicon Primer Panels for Diagnostic of SMoV and SVBV

Tiled amplicon PCR (TA-PCR) primers were designed based on a set of complete genomes of SMoV and SVBV present in the NCBI database. Only complete genomes were considered for analysis (Supplementary Tables S2–S4). The genomes of each species were aligned using Clustal Omega by Geneious Prime (version 2021.1.1, https://www.geneious.com/ (accessed on 23 May 2024)). Multipartite viruses had each component of their genome aligned separately. The final alignment was submitted to the web-based service of PrimalScheme (https://primalscheme.com/, accessed on 23 May 2024). Tiled amplicons were designed to be around 500 base pairs (bp) long, allowing for efficient sequencing with minimum overlap of 75 bases between tiles, with length between 20 and 30 nucleotides and annealing temperature around 65 °C. Primer panels were designed to cover most of the genome with the minimum number of tiles. The primers were grouped in two pools for amplification to avoid long amplicons.

**Figure 1:**
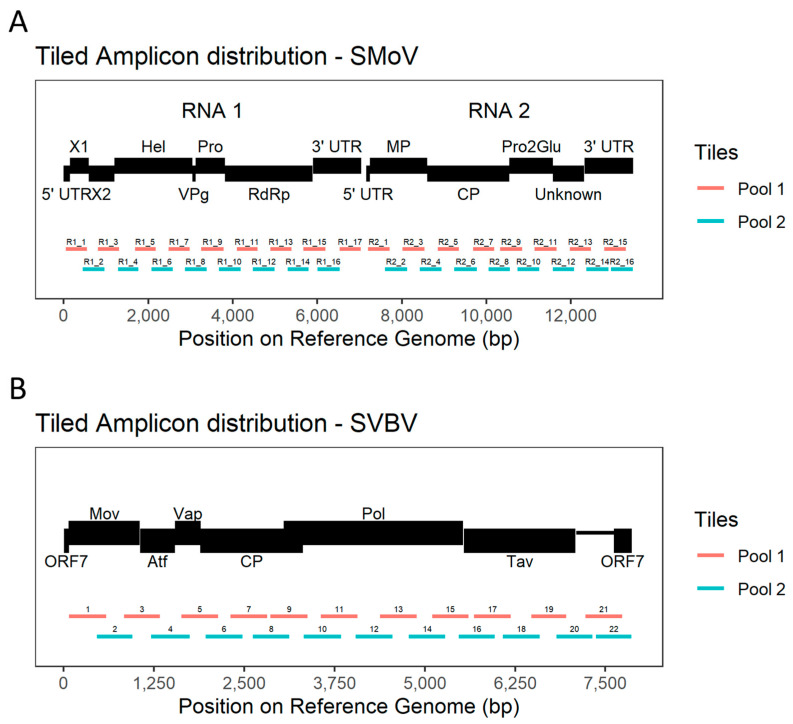
Tiled amplicons were designed to cover most of the bipartite (single-stranded RNA1 and RNA2) strawberry mottle virus (SMoV) and strawberry vein banding virus (SVBV) genomes. For ease of depiction, the genome of SVBV circular double-stranded DNA (dsDNA) is shown here as linear. Reference genomes—SMoV (NCBI accessions LC550285 and LC550286) and SVBV (NCBI accession NC_001725). (**A**)—Genomic structure of SMoV and distribution of tiled amplicons. RNA 1 consists of untranslated regions at the 5′ (5′ UTR) and 3′ (3′ UTR) extremities, and a single Open Reading Frame (ORF) P1 which, when cleaved, releases the mature proteins of X1, X2, Helicase (Hel), viral genome-linked protein (VPg), 3C-like protease (Pro), and an RNA-dependent RNA polymerase (RdRp). RNA2 has 5′ UTR and 3′ UTR flanking the ORF for the P2 polyprotein that generates a movement protein (MP), capsid protein (CP), Pro2Glu and a suppressor of RNA silencing protein (P28). (**B**)—The SVBV genome has 7 ORFs encoding for a movement protein (Mov), an aphid transmission factor (Atf), a virion-associated protein (Vap), a capsid protein (CP), a polymerase polyprotein (Pol), a translational trans-activator/inclusion body matrix protein (Tav), and ORF 7.

Primers were then tested in silico by Geneious Prime (version 2021.1.1, https://www.geneious.com/ (accessed on 23 May 2024)) for the amplification of the respective tiles from the aligned genomes of each virus, assuming a maximum of five mismatches and an amplicon size of 500 bp. Coverage gaps due to sequence diversity in certain groups of strains were minimised by resubmitting the divergent strains in a new alignment to PrimalScheme. The newly designed primers were then compared and equivalent tiles from it were added to the correspondent primer pool. The new set of primers (Figure 1) was then tested again in silico for amplification of the viral genome, assuming five mismatches and amplicon size of 500 bp. Final primer sets were considered ready when most of the viral genome was covered by tiles, independently of strain (Supplementary Tables S7 and S8).

### 2.7. Tiled Amplicon PCR

Each primer was reconstituted in low EDTA TE buffer to produce a primer stock of 100 µM. Two pools of primer stocks from alternate regions were prepared for each viral target, combining an equal volume of each primer stock into its respective pool. The final pools had a combined molarity of 100 µM and were used as the primer working solution.

The optimisation of the amplification step of tiled amplicons was performed in stages using positive control samples for each virus. Complementary DNA (cDNA) was produced from 100 ng of total RNA using the SuperScript III First-Strand Synthesis System (ThermoFisher Scientific) and ProtoScript II First Strand cDNA Synthesis kit (New England Biolabs) following manufacturer instructions. The resulting single-stranded cDNA was then used as template for the TA-PCR using the KAPA HiFi Hotstart ReadyMix PCR kit (Roche, Cape Town, South Africa) following the recommended conditions for initial denaturation, cycle denaturation, and final extension times and temperatures, but performing the annealing step at 65 °C for 30 s followed by extension at 72 °C for 5 min, upon 40 amplification cycles. Titration of individual primer concentrations in the final reaction was used to determine the optimal concentration for each primer. Once primer concentration was optimised, amplification was tested using the following PCR master mixes: KAPA HiFi HotStart ReadyMix PCR Kit (Roche), Phusion U Multiplex PCR Master mix, Platinum Multiplex PCR Master mix and), Qiagen Multiplex PCR kit (Qiagen, Hilden, Germany), and Q5 High-Fidelity 2X Master Mix (New England Biolabs). Amplification was performed with initial denaturation at 98 °C for 2 min, followed by 40 cycles of 98 °C for 10 s, 65 °C for 30 s, and 72 °C for 5 min and a final extension step at 72 °C for 7 min. The final amplification protocol, including amplification cycle and master mix, was chosen based on producing a single, strong visible band of amplicons of the expected size by gel electrophoresis (around 500 bp), followed by ONT sequencing of the amplicons to confirm expected genome amplification.

The TA-PCR reaction consisted of 2.5 µL of the cDNA, 12.5 µL Q5 High-Fidelity 2X Master Mix (New England Biolabs), one of the primer pools targeting a virus with a final concentration of 0.18 µM for individual primers, and volume adjusted to 25 µL. Each sample was amplified by each pool in separate reactions, and upon conclusion, both reactions were mixed to a total of 50 µL.

### 2.8. Simplex Sequencing of Tiled-Amplicon Samples

PCR cleanup was carried out using AMPure XP beads (Beckman Coulter, Mount Waverley, VIC, Australia) with a bead/sample ratio of 0.8:1 *v*/*v*. Purified amplicons were quantified using Qubit 1X dsDNA HS Assay Kit (Thermo Fisher Scientific). The Ligation Sequencing Kit SQK-LSK112 (Oxford Nanopore Technologies, Oxford, UK) was used for library preparation of simplex tiled-amplicon sequences, with additional enzymes. Briefly, 100 fmol of amplicons (500 ng for 500 bp amplicons) were end-repaired using the NEBNext Ultra II End-repair/dA-tailing Module and NEBNext FFPE DNA Repair Mix (New England Biolabs). A 23.5 µL normalised aliquot of amplicon was added to 0.5 µL DNA Control Sample, 1.75 µL NEBNext FFPE DNA Repair Buffer, 1 µL NEBNext FFPE DNA Repair Mix, 1.75 µL Ultra II End-prep Reaction Buffer, and 1.5 µL Ultra II End-prep Enzyme to a final reaction volume of 30 µL and incubated at 20 °C for 5 min, with denaturation of enzymes at 65 °C for 5 min. AMPure XP bead clean-up was performed using bead/sample ratio of 0.8:1 *v*/*v*, and eluted in 30 µL of nuclease-free water. Adapter Ligation was obtained by using 30 µL of end-repaired DNA, 12.5 µL Ligation Buffer, 5 µL of Adapter Mix H, 10 µL Quick DNA Ligase and incubated at 20 °C for 10 min. AMPure XP bead clean-up was performed using bead/sample ratio of 0.4:1 *v*/*v*,; beads were washed with Short Fragment Buffer and the library eluted in 7 µL of Elution Buffer. Sequencing of these libraries was performed on Flongle cells FLO-FLG001 (Oxford Nanopore Technologies), using the MinKNOW (version 23.04.6, https://nanoporetech.com (accessed on 23 May 2024)) using a High-Accuracy model (400 bps) on a Linux-based Ubuntu 18.04.6 LTS operating system.

### 2.9. Multiplex Sequencing of Tiled Amplicon Samples

The Native Barcoding Kit SQK-NBD114.24 (Oxford Nanopore Technologies) was used for library preparation of multiplex tiled amplicon sequences, with additional enzymes. Every run included a set of samples to be tested plus a water control for assessment of contamination throughout the library preparation. End-repair of 100 fmol of amplicons (65 ng for 500 bp amplicons) was carried out using Next Ultra II End-repair/dA-tailing Module (New England Biolabs). Amplicons were normalised to 6.7 µL and combined with 0.5 µL Diluted DNA Control Sample (DCS), 0.9 µL Ultra II End-prep Reaction Buffer, and 0.4 Ultra II End-prep Enzyme to a final reaction volume of 8.5 µL and incubated at 20 °C for 5 min, denaturation of enzymes at 65 °C for 5 min. AMPure XP bead clean-up was performed using bead/sample ratio of 0.8:1 *v*/*v*, and elution in 5 µL of nuclease-free water. Barcode ligation was performed with 3.75 µL end-repaired DNA, 1.25 µL native barcode, and 5 µL Blunt/TA Ligase Master Mix (New England Biolabs), incubated for 20 min at 20 °C; all reactions for a given run were pooled together and AMPure XP bead clean-up was performed using bead/sample ratio of 0.8:1 *v*/*v*, with barcoded DNA eluted in 17.5 µL of nuclease-free water. Adapter Ligation was obtained by using 15 µL of barcoded DNA, 2.5 µL of Native Adapter, 5 µL of Next Quick Ligation Buffer 5x (New England Biolabs), and 2.5 µL Quick DNA Ligase, incubated at 20 °C for 20 min. AMPure XP bead clean-up was performed using bead/sample ratio of 0.8:1 *v*/*v*; beads were washed with Short Fragment Buffer and library eluted in 7 µL of Elution Buffer. Sequencing of these libraries was performed on Flongle cells FLO-FLG114 (Oxford Nanopore Technologies), using the same software as described above.

### 2.10. Data Analysis of Samples Obtained for TA-PCR

The multipartite reference genome of SMoV was concatenated into a single fragment to facilitate analysis. The fastq files with passed reads obtained for the tiled amplicons were concatenated into a single file. Reads with total length between 400 bp and 900 bp were selected using SeqKit (version 2.2.0, https://github.com/shenwei356/seqkit (accessed on 23 May 2024)) and were mapped to the reference genomes of the correspondent targets of the TA-PCR assay using minimap2 (v.2.24, https://github.com/lh3/minimap2 (accessed on 23 May 2024)). Mapped reads had a minimum mapping quality of 10. Mapped reads were sorted according to threshold conditions using SAMtools (v.1.15.1). A draft consensus was generated by BCFtools (v.1.5.1) and identified by BLAST+ as described previously. TaxonKit (version 0.13.0, https://github.com/shenwei356/taxonkit (accessed on 23 May 2024)) was used to produce human readable tables of draft consensus.

## 3. Results

### 3.1. Limit of Detection of RT-qPCR Assays Targeting SMoV and SVBV

The RT-qPCR assays used for quantification of SMoV and SVBV were assessed for their limit of detection (LoD) expressed in copy numbers using a serial dilution of synthetic RNA transcripts including the amplicon sequence generated by the test in question. The synthetic RNA transcript used to prepare the RNA standard panels included the region flanking the binding sites for primers and the probe binding site.

**Figure 2:**
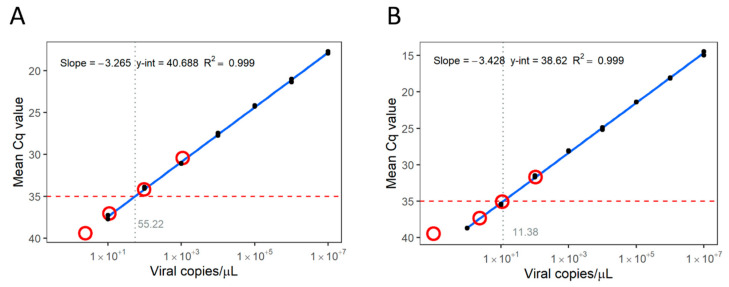
The limit of detection (LoD) of RT-qPCR for assays used for the detection of strawberry mottle virus (SMoV) and strawberry vein banding virus (SVBV) was calculated using synthetic RNA transcripts. (**A**)—The estimated LoD of the assay to detect SMoV is 55.82 viral copies/µL (grey dotted vertical line) at a quantification cycle (Cq) of 35 (red horizontal dashed line). (**B**)—The estimated LoD of the assay to detect SVBV is 11.38 viral copies/µL (grey vertical dotted line) at Cq = 35 (red horizontal dashed line). Red circles indicate dilutions of positive control samples used to assess the sensitivity of HTS-based diagnostics.

The cRNA standard panel for absolute quantification of SMoV used an RNA transcript of 382 nt long and was tested over a linear dynamic range of 1 × 10^1^ to 1 × 10^7^ viral copies/µL (R^2^ = 0.999, efficiency = 102%, Figure 2A). At Cq = 35, the LoD of this test was determined to be 5.52 × 10^1^ viral copies/µL. Despite the DNA nature of the SVBV genome, we used the same RT-qPCR approach for detection as RNA transcripts are important components of the caulimovirid life cycle and can logically be used for their detection. An RNA transcript of 379 nt was tested against the same range of concentrations as above (R^2^ = 0.999, Efficiency = 95.8%, Figure 2B). At Cq = 35, the LoD of this test was 1.14 × 10^1^ viral copies/µL.

Using these data, samples for testing the LoD of HTS-based methods were prepared to detect SMoV at concentrations ranging from 1 × 10^0^ viral copies/µL (Cq = 39.42, 2.44 × 10^0^ viral copies/µL) to 1 × 10^3^ viral copies/µL (Cq = 30.42, 1.39 × 10^3^ viral copies/µL), and SVBV from 1 × 10^−1^ viral copies/µL (Cq = 39.46, 5.70 × 10^−1^ viral copies/µL) to 1 × 10^2^ viral copies/µL (Cq = 31.69, 1.05 × 10^2^ viral copies/µL).

### 3.2. Metatranscriptomic Approach and RT-qPCRs Can Detect Viruses in Similar Levels

The first approach to HTS-based diagnostics of viruses involved sequencing ribo-depleted samples using Illumina paired-end sequencing (150 bp). The sequencing data received from the sequencing facility were tested for quality and subsampled in triplicates using different seed values (11, 17, and 23) for each replicate. The subsampling was conducted to obtain final files with 3 M, 7 M, 15 M, 30 M, 40 M, and 60 M PE reads (Supplementary Tables S5 and S6). The original file with 120 M PE reads was also analysed for each sample sent for sequencing.

**Figure 3:**
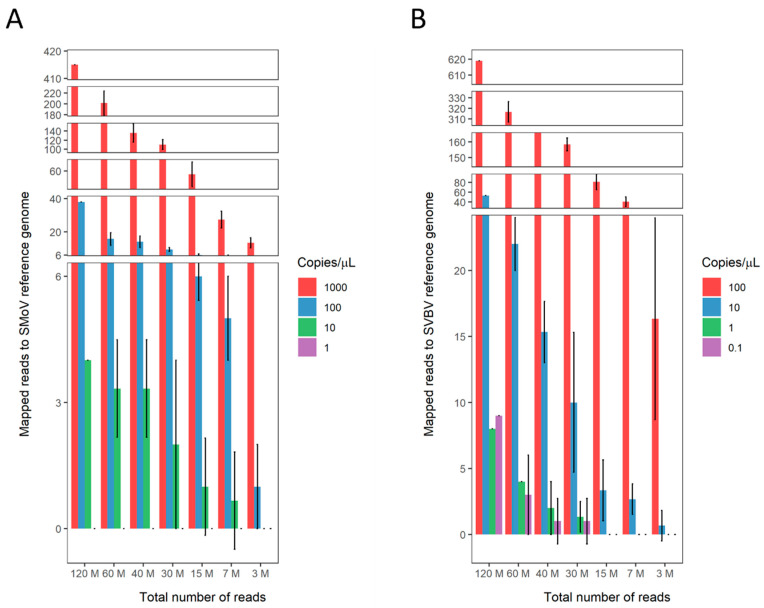
Number of reads mapped (M for million) to reference genome of viruses obtained from different sequencing subsampled depths for dilutions of positive control samples. (**A**)—Reads mapped to the strawberry mottle virus (SMoV) reference genome for samples with 1 to 1000 viral copies/µL. (**B**)—Reads mapped to strawberry vein banding virus (SVBV) reference genome for samples with 0.1 to 100 viral copies/µL. Standard deviation is represented by black bars.

Four SMoV samples were sequenced using the Illumina NovaSeq sequencing platform. SMoV sequences were successfully mapped to the reference genome in the three most concentrated samples (Figure 3A). In the case of the sample with 10 viral copies/µL, viral sequences were consistently detected in datasets of 120 M, 60 M, and 40 M PE reads but further subsampling gave an inconsistent number of mapped reads between replicates (Figure 3A). Mapping the data obtained for these samples to the SVBV reference genome did not return any matches.

All four SVBV samples ranging from 1 × 10^−1^ to 1 × 10^2^ viral copies/µL generated reads that could be mapped to the reference genome of this virus (Figure 3B). Subsampling of the initial datasets was consistent for detection of 10 viral copies/µL when 120 M, 60 M, and 40 M PE reads were analysed. A single viral copy/µL was detectable in datasets with 120 M and 60 M PE reads. Further dilutions or subsets of the initial datasets were not effective in detecting this virus (Figure 3B). No reads mapped to the SMoV reference genome.

**Figure 4:**
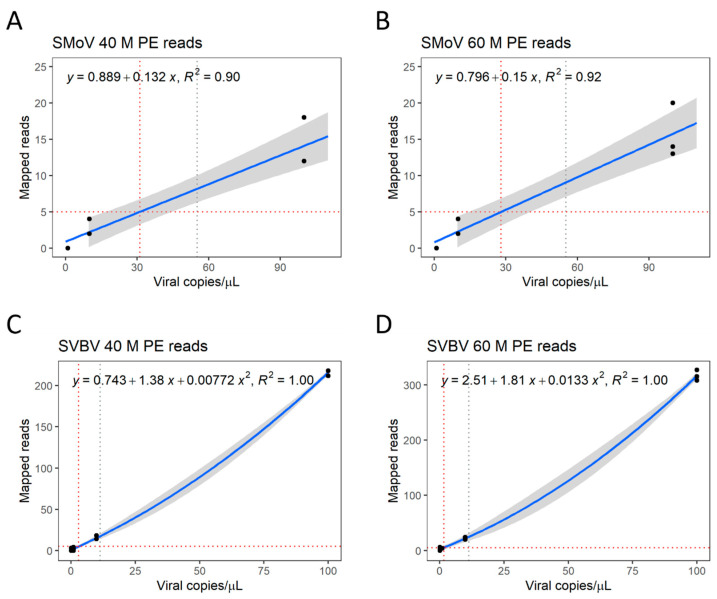
Estimated limit of detection (LoD) of metatranscriptomics to detect viruses. Regression of mapped reads in proportion to viral copies of strawberry mottle virus (SMoV) from datasets with 40 M paired-end (PE) reads (**A**) and 60 M PE reads (**B**), and strawberry vein banding virus (SVBV) from datasets of 40 M PE reads (**C**) and 60 M PE reads (**D**), respectively. A minimum threshold of 5 reads mapping to a reference genome was established. Grey dotted line represents LoD of respective RT-qPCR. Red dotted lines represent intersection between minimum mapped read count threshold and viral copy number. Grey areas represent confidence at *p* > 0.95.

A minimum of five reads mapping to the reference genome of a given virus was defined as the threshold to consider whether a sample was positive or not, requiring further assessment by other methods, such as PCR. This threshold represents an average of mapped reads greater than the standard deviation observed when data were subsampled. Consequently, regression of the data obtained from subsampling the datasets to 40 M and 60 M PE reads allows the detection of SMoV in samples with viral copy numbers as low as 3.11 × 10^1^ and 2.80 × 10^1^ viral copies/µL, respectively (Figure 4A,B). The SVBV datasets with 40 M and 60 M PE reads have their LoD at 3.03 × 10^0^ and 1.36 × 10^0^ viral copies/µL, respectively (Figure 4C,D). For both viruses, these numbers represent a similar range of detection to that observed when RT-qPCR is performed.

### 3.3. Genomes of SMoV and SVBV Are Successfully Amplified Using Custom Single-Target and Multi-Target TA-PCR Assays

**Figure 5:**
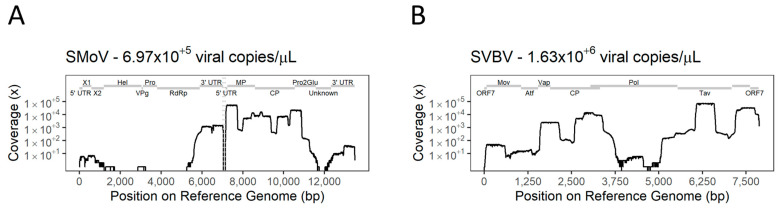
Nucleotide coverage of reference genomes upon use of single-target tiled amplicon PCR (TA-PCR) custom primer sets of strawberry mottle virus (SMoV) (**A**) and strawberry vein banding virus (SVBV) (**B**) present in positive control samples. Genomic domains of each virus are displayed by grey bars.

Most of the genome of SMoV was successfully amplified by single-target TA-PCR and sequenced using Flongle flow cells (Oxford Nanopore Technologies). Sequencing of the tiled amplicons obtained for a positive control sample of SMoV (Cq = 21.61, 6.97 × 10^5^ viral copies/µL) was able to generate up to 148,060 reads, spanning 65.17% of the reference genome sequence (Figure 5A). Despite optimisation, the primer panel designed was not able to provide a good coverage of the RNA 1 of SMoV. The best coverage was observed in the genomic regions encoding for the movement protein (MP), capsid protein (CP), Pro2Glu of RNA1 segment, and the untranslated region at the 3′ arm of each RNA segment, with reference coverage above 1000 times. The identity of the organism was validated by accessing the identity of the consensus sequence obtained from a single sample by comparison to NCBI.

A positive control sample of SVBV (Cq = 17.32, 1.63 × 10^6^ viral copies/µL) was also amplified by TA-PCR and 96.07% of the reference genome was recovered from the 194,942 reads obtained (Figure 5B). The best covered regions of the genome of SVBV encoded the inclusion body matrix protein (Tav), open reading frame 7 (ORF7), capsid protein (CP), and vector-associated protein (Vap). Blastn analysis of the consensus sequences obtained from mapping the reads to the reference genome confirmed that they originated from the correct virus sequence.

**Figure 6:**
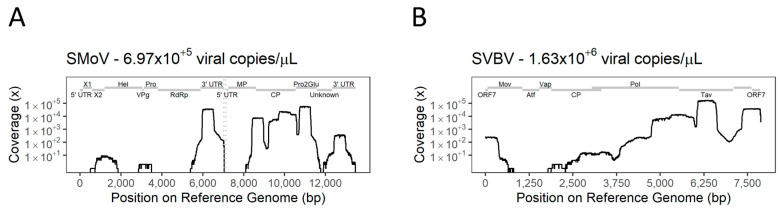
Coverage of reference genomes by multiple-target tiled amplicon PCR (TA-PCR) custom primer sets retrieved for strawberry mottle virus (SMoV) (**A**) and strawberry vein banding virus (SVBV) (**B**) present in positive control samples. Genomic domains of each virus are displayed by grey bars.

As amplification for most of the target genomes was obtained for both viruses in single-target assays, the respective primer pools were combined to assess the amplification of multiple targets in a single reaction. The positive controls for SMoV and SVBV used for the single-target testing were used to evaluate the amplification using the multi-target primer pools. The PCR amplification step was re-tested using different single primer concentrations in the final reaction, but the optimised conditions for single-target assays were also the best performing ones for the multi-target approach. The multi-target TA-PCR allowed the recovery of 62.74% of the genome of SMoV from 171,521 reads, and 82.48% of the SVBV genome from 331,234 reads (Figure 6A and B respectively). The distribution of coverage was like those observed with single-target TA-PCR, although some minor exceptions were noted, including a decrease in the coverage of MP (SMoV) and Vap and CP (SVBV), and an increase in coverage of the polymerase polyprotein Pol (SVBV).

### 3.4. Multi-Target TA-PCR Assay Has LoD Similar to That of qPCR Assays

Given the extended coverage of the reference genomes observed when positive control samples were assessed using the proposed multi-target TA-PCR approach, we wanted to assess the ability of the method to multiplex samples at a range of concentrations. The protocol was, therefore, used to generate data from the 10-fold serial dilutions of SMoV and SVBV to determine the LoD expressed in viral copies/µL for each virus.

**Figure 7:**
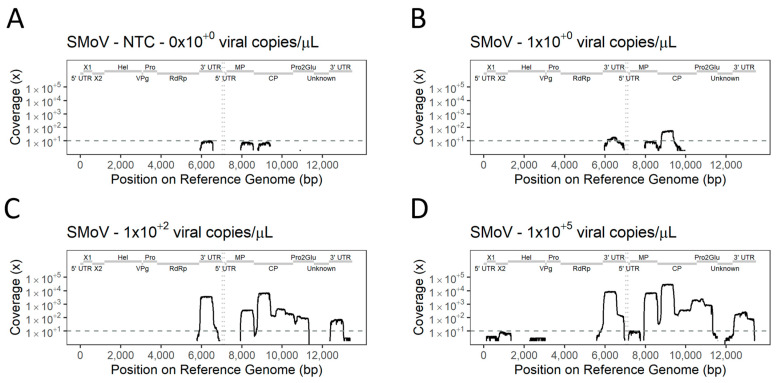
Whole genome amplification of strawberry mottle virus (SMoV) using a multiple-target TA-PCR primer set across samples with various viral titres. (**A**)—Coverage of reference genome by non-template control (NTC) used to assess contamination and determine a flexible coverage threshold for detection in each experiment. (**B**–**D**)—Coverage of reference genome by amplicons obtained from different dilutions of positive control material. Grey dashed line represents the flexible threshold of coverage (threshold variable between experiments). Fixed threshold of coverage (threshold = 10) coincided with flexible coverage threshold in this experiment. Genomic domains of the virus are displayed by grey bars on top of each graph.

**Figure 8:**
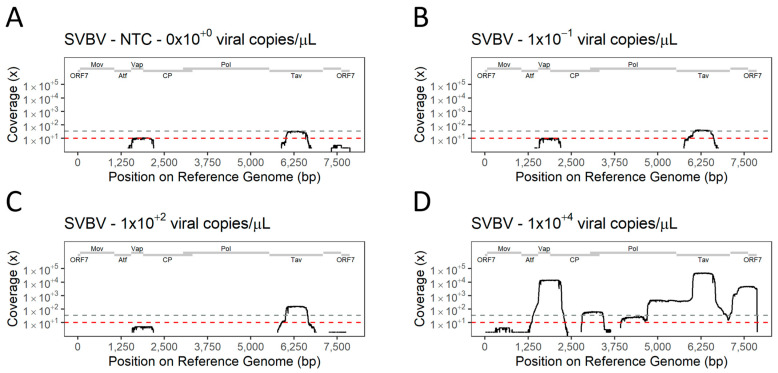
Amplification of the whole genome of strawberry vein banding virus (SVBV) from samples with various viral titres using a multi-target tiled amplicon PCR. (**A**)—Coverage of reference genome by non-template control (NTC) was used to assess contamination and determine a flexible coverage threshold for detection in each experiment. (**B**–**D**)—Coverage of reference genome by amplicons obtained from different dilutions of positive control material. Red dashed line represents the fixed threshold of coverage (threshold = 10). Grey dashed line represents the flexible threshold of coverage (threshold variable between experiments). Genomic domains of the virus are displayed on grey bars on top of each graph.

Sequences mapping to the reference genomes were obtained from all samples, including the NTC. Partial genomes from both SMoV (Figure 7) and SVBV (Figure 8) were successfully sequenced from multiplex library preparations. The multi-target approach was tested by performing TA-PCR, library preparation, and sequencing in five independent runs for SMoV (Supplementary Table S9) and six independent runs for SVBV (Supplementary Table S10). The number of reads mapping to the reference genome, and the reference coverage given by the data were proportional to the number of viral copies in the tested samples. A low proportion of reads mapped to target references absent in the samples. As the mapping of reads to the reference genome of the target organisms was not enough to differentiate between positive and negative detection, we established two groups of criteria to allow a qualitative classification of the mapped data.

**Figure 9:**
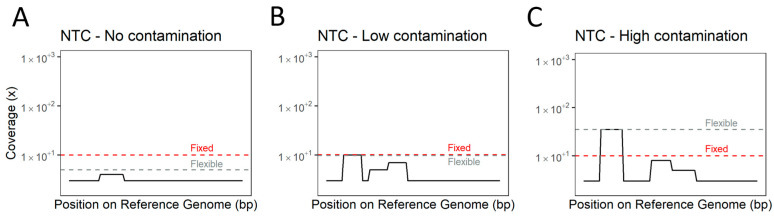
Use of non-template control (NTC) to determine the flexible threshold depending on the level of cross-contamination inferred by mapping of reads from NTC. (**A**)—No contamination might eventually still have a few reads mapping to the reference genome but allows flexible threshold to be lower than fixed threshold. (**B**)—Fixed and flexible thresholds may have similar values upon low levels of cross-contamination between samples in the same run. (**C**)—High levels of cross-contamination between samples are compensated by higher flexible threshold to mitigate false positives.

The initial approach, a fixed threshold, considered that a positive sample should have a minimum coverage of the reference genome of two tiles long (1000 bp) at a minimum depth of 10. Using this approach, the results obtained for NTC are interpreted as an indicator of contamination. The second approach, the NTC-based flexible threshold, considered that the minimum depth of coverage of the reference genome should be the maximum depth obtained for the NTC sample for a single nucleotide, and this should be achieved for at least one tile length (500 bp) for other tested samples. In this approach, the NTC was used to estimate the contamination during laboratory procedures and is variable between runs. The lowest defined level for the flexible threshold was set at a sequencing depth of five times (Figure 9).

**Figure 10:**
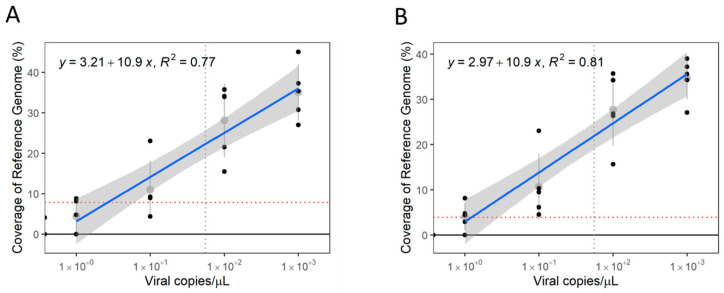
Limit of detection of multi-target tiled amplicon PCR of strawberry mottle virus (SMoV) using different types of reference coverage threshold. (**A**)—Fixed threshold of 2-tile-long reference coverage (red dotted line). (**B**)—Flexible threshold based on non-template control (NTC) sample (red dotted line). Grey dotted lines represent LoD of RT-qPCR assay for SMoV diagnostics. Grey areas represent confidence at *p* > 0.95. Grey dots represent average and grey bars the standard deviation.

**Figure 11:**
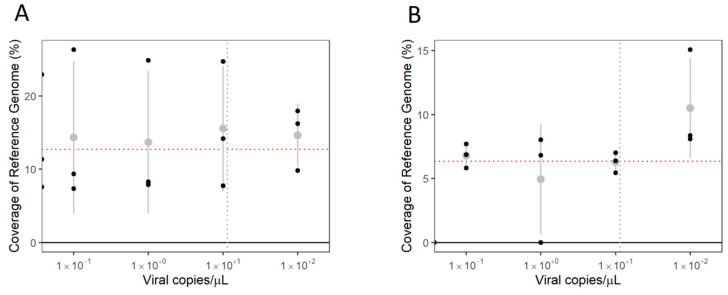
Limit of detection of multi-target tiled amplicon PCR of strawberry vein banding virus (SVBV) using different types of reference coverage threshold. (**A**)—Fixed threshold of 2-tile-long reference coverage (red dotted line). (**B**)—Flexible threshold based on non-template control (NTC) sample (red dotted line). Grey dotted lines represent LoD of RT-qPCR assay for SVBV diagnostics. Grey dots represent average and grey bars the standard deviation.

Both fixed and flexible thresholds were able to define the minimum coverage of reference genome as a parameter for differentiating positive samples from negative samples. SMoV (Figure 10) and SVBV (Figure 11) were detected in levels down to 10 viral copies/µL when either threshold was used. Nevertheless, the use of the fixed threshold allowed the NTC samples of some sequencing runs to be considered false negatives, with coverage of reference genomes very close to those of samples at the LoD. The flexible threshold was able to reduce the noise caused by cross-contamination between samples without reducing the sensitivity of the assay, assuming that the level of contamination is that observed for the NTC sample.

The coverage of reference genomes of the viruses assessed by TA-PCR was significantly impacted by low viral copy numbers in the samples. Even so, the most prominent areas amplified from samples with high viral content were still recovered in diluted samples. The samples with low viral copy numbers of SMoV had the coverage of the reference genome restricted to the 3′ UTR portion of the RNA 1 and portions of the regions encoding MP and CP on RNA2. SVBV samples had the areas encoding for Vap and Tav as the only areas amplified at low viral copy numbers.

## 4. Discussion

The development and validation of two HTS enrichment-based protocols for detection of plant viruses was carried out for samples with low viral titre as alternatives to the standard quantitative PCR-based methods of detection. The RNAseq and TA-PCR protocols both demonstrated sensitivity as low as 10 viral copies/µL of SMoV and SVBV, comparable to qPCR-based methods. In facilitating simultaneous multiple detection and sequence characterisation of viruses, HTS methods offer clear advantages over older PCR-based techniques.

Despite the quantitative nature of qPCR and its variations, the final purpose of its use in diagnostics is the assignment of a qualitative result, either confirming or rejecting the infection status of a given sample [11]. Comparing the analytical sensitivity of diagnostic methods requires the assessment of the LoD of a standard assay based on absolute or relative quantification of its targets. The use of Cq as a unit to assess sensitivity is not recommended because several factors can contribute to variations between samples, such as sample matrices, reagent batches, and operators [36]. These variations can be normalised when a set of standards of known concentration are used to translate these Cqs into quantifiable units [37,38]. Multiple reports comparing the use of HTS and qPCR usually employ artificial measurements for sensitivity based on qualitative rather than analytical considerations [36]. The use of ‘copy number per volume’ allows a comparison of the (analytical) sensitivity of qPCR assays to HTS-based methods with completely different input amounts of TNA, minimising experimental and analytical interferences not directly related to the number of targets in the test. Although rarely used when comparing detection methods for plant pathogenic viruses, such as those done for cucumber green mottle virus (CGMMV) [31], this approach is a standard feature on reports about new assays for diagnostics of viruses of medical importance, allowing easier and direct comparison between methods and reports [26,39,40].

Agreement on virus detection between qPCR and RNAseq results was shown as proof of equivalence (diagnostic sensitivity) for these methods in several reports [15,24,25,31,41,42,43,44,45,46,47,48]. However, most of these reports had RNAseq performed with naturally infected samples whose viral copy were either not assessed, or their viral titre was much higher than the expected LoD of the qPCR assays used, as evidenced by low Cq numbers. The abundance of sequences of interest in these samples combined with the low sequencing depth used in some of these studies may lead to the assumption that RNAseq is not as sensitive as PCR when low titre samples are tested. Some of these studies tried to address this problem by preparing dilution series covering a limited range of viral titres, although not reaching the LoD of the test [44,48]. Experimental validation of a new protocol may be conducted with low titre samples bordering the LoD of a reference test. Challenging a new HTS test to its analytical limits allows the proper assessment of test conditions, such as adequate sequencing depth and suitable enrichment methods for unbiased detection [18,49]. Although high sequencing depth datasets can still be expensive when a single virus is considered for the analysis, screening for multiple organisms can make ribodepleted total RNA sequencing competitive. We challenged both qPCR and metatranscriptomic approaches with low titre samples to determine the minimum sequencing depth required to accurately detect viruses at the same level as qPCR. We observed that raw datasets with 40 M to 60 M PE reads were consistent for detection of both SMoV and SVBV at the LoD of their respective standard qPCR-based methods of detection. Our findings corroborated the reports obtained by other groups that investigated similar sensitivity-based methodology using RNAseq, in which at least 30 M reads were required per sample to confirm the diagnostic results obtained by qPCR at their LoD from animal, human, and environmental samples, normally ranging between 1 to 1000 viral copies/µL [39,50,51,52].

The inclusion of controls is an important tool used to assume that results obtained from HTS data sets have acceptable levels of false positives and false negative results [15,18]. The assays presented here were conducted with positive controls themselves, as the presence and concentration of all targets was determined before HTS techniques were applied. A positive control sample prepared for one virus could also be considered the negative control for the other virus, being that they were processed together. Guidelines developed by working groups describe a set of steps to determine contamination and the need for controls in HTS assays [19,20,22,23]. According to these guidelines, our proposed RNAseq workflow offers tools to assess the ability to detect the expected targets (number of generated reads and read identity), discrimination of false negatives and false positives (RNA quality, sequencing depth and read identity), variant filtering (mapping quality) and inconclusive results using either bioinformatics or RT-qPCR. The non-adoption of an alien control spiked in all samples did not allow for further monitoring of sequencing performance [22]. The use of a parallel sample with an alien target could offer a better estimation of dynamic and hard-to-predict sources of inconclusive results, such as cross-sample contamination and barcode-hopping [20,48,52]. The RNAseq method developed in this study will be used as a tool to accelerate the screening for strawberry plants held in plant quarantine. No changes to sample collection, bulking, subsampling, and NA extraction methods will be necessary as the sensitivity of RNAseq is equivalent to that of the current PCR-based methods used in our facility. Any reads found to match the identity of any target of interest will be further investigated using RT-qPCR assays with similar analytical sensitivity and validated to the known variability of the target species [12,47].

TA-PCR is a cheaper and effective alternative to ribodepleted RNA sequencing if the target of interest is known. Whole genome amplification using tiled amplicons has been primarily employed for epidemiological studies of viruses with medical importance, such as Zika [27], SARS-CoV-2 [30,53,54], and orthohantaviruses [40], from clinical or environmental samples. Applications involving plant pathogenic viruses have been reported for cucumber green mottle mosaic virus (CGMMV) [31,55] and tomato brown rugose fruit virus (ToBRFV) [32]. We also investigated the use of TA-PCR to enrich viral target sequences from TNA samples. Although whole genome amplification of plant pathogenic viruses by TA-PCR has been tried by other groups for a single virus in a single assay, the technique could be extended to more viral targets. Here, we present the first report of TA-PCR being used to detect, simultaneously, two viruses with distinct genome replication strategies in the same assay: SMoV is a single-strand RNA (ssRNA) virus, and SVBV is a double-strand DNA (dsDNA) virus.

Applying TA-PCR to detect both SMoV and SVBV, we observed a significant reduction in reference genome coverage as samples were successively diluted towards the LoD of the qPCR assays in proportion to the low viral titre analysed. Despite not covering the whole genome of the targets, TA-PCR still allowed for accurate detection of viruses at low titre and provided sequence information from highly expressed genes of the target viruses that can be used for confirmation of amplicon hits. These results contrast with the whole genome amplification to detect CGMMV, in which the virus genome was sequenced completely even from samples with as little as 15 viral copies/µL [31]. Reports using TA-PCR on screening for pathogenic viruses from a variety of hosts reported low genome coverage for these organisms when copy numbers were low in the tested samples, in a similar manner as observed in our data [27,53,56]. However, most reports of TA-PCR assays did not aim to use this technique as diagnostic method as we propose here. Considering that the aim of a diagnostic test is to determine the presence or absence of a given pathogen, we provided a set of parameters to analyse the partial coverage of the targeted organisms obtained by TA-PCR for an accurate and specific detection of these viruses.

Cross-contamination has been reported in other studies using TA-PCR for whole genome amplification of viruses and we also observed this in our data for a few sequencing runs. Contamination could be a result of mistakes during the large number of manual pipetting steps, amplicon contamination from previous experiments, or even barcode hopping during sequencing [26,27,53]. As a consensus, the use of controls to measure contamination or mitigate its occurrence are essential for the accurate use of TA-PCR or any other technique for diagnostics. We propose the use of a NTC as a sentinel to indicate the general level of contamination between samples throughout the whole protocol, from RT-PCR to data analysis. Fixed and flexible thresholds for reference genome coverage were compared to determine whether the viruses can be detected in low viral titres while accounting for the occurrence of false positives. The use of the highest coverage for a single nucleotide in NTC samples as the minimum threshold for reference coverage reduced the occurrence of false positives maintaining the sensitivity of the test. Both viruses targeted by the assay were separately analysed and distinct thresholds assigned to each of them. The presence of a sample with high copy number of a particular virus was associated with higher levels of contamination within a run for that particular virus in our data sets and in other reports [26,57].

In theory, PCR-based diagnostics are capable of identifying pathogens from as little as three target copies [58]. However, several factors can influence the outcome of PCR results, including chemical composition of the samples, primer-binding kinetics due to changes in the target sequence, accurate subsampling whilst spiking reactions with testing sample, and so on. Therefore, most PCR assays require multiple starting molecules for the effective detection of a target [36]. To overcome the limitation of the number of starting molecules, especially from samples with low viral titre, amplicon sequencing of more abundant viral targets might perform better than TA-PCR. ORFs normally expressed in higher levels, such as those encoding for capsid protein, movement proteins, and proteins related to the direct interaction to the host, have already been used successfully for this purpose for detection of plant pathogenic viruses, such as for prunus necrotic ringspot virus in trees [59], cucumber mosaic virus, pea early browning virus, bean yellow mosaic virus, and pea seed-borne mosaic virus in grain crops [60] in Australia and 34 viruses and viroids in *Malus* and *Prunus* in the United States [61]. Yet, the lack of information regarding the LoD expressed as viral copies per volume of TNA does not allow for direct comparison of these reports to the present study in terms of sensitivity and reproducibility.

We present, here, two distinct and effective methods for the enrichment of plant pathogenic virus sequences from samples with low viral titre. Both enrichment strategies, RNAseq and TA-PCR, allowed for the accurate detection of viruses, with distinct replication strategies, at similar levels to those observed for PCR-based detection. The RNAseq approach, although more expensive, has the flexibility for detection of known and unknown targets in samples with low viral titre according to local regulation, requiring minimal adjustments to be applied to other commodities. With the reduction in costs for HTS platforms, this method may become as cheap as qPCR in a matter of a decade and might change the way diagnostics, as we know it today, works. TA-PCR offers a cheaper targeted approach useful for genome characterisation of regulated viruses based on low depth sequencing. As described for other targeted viruses, successful use of tiled amplicons for diagnostics in the long term might require constant surveillance and updates on new variants and the development of accessory primer panels to account for any changes.

We have described the first report on the detection of multiple plant viruses using a single TA-PCR assay, even targeting viruses with different genome composition. This approach has potential use not only for post-entry quarantine, but also in border and general surveillance programmes in which there is a defined list of targets and for any application where high-sensitivity diagnostics combined with simultaneous sequence characterization is required.

## Data Availability

Data are contained within the article and Supplementary Materials.

## Supplementary Materials

The following supporting information can be downloaded at: https://www.mdpi.com/article/10.3390/v16101550/s1, Table S1: Primers used in this study; Table S2: References genomes of strawberry mottle virus (SMoV) RNA1 used for design of primer; Table S3: References genomes of strawberry mottle virus (SMoV) RNA2 used for design of primer; Table S4: References genomes of strawberry vein banding virus (SVBV) used for design of primers; Table S5: Summary of RNAseq data of strawberry mottle virus (SMoV); Table S6: Summary of RNAseq data of strawberry vein banding virus (SVBV); Table S7: Primer panel for tiled amplicon PCR of strawberry mottle virus (SMoV); Table S8: Primer panel for tiled amplicon PCR of strawberry vein banding virus (SVBV); Table S9: Summary of tiled amplicon PCR data for strawberry mottle virus (SMoV); Table S10: Summary of tiled amplicon PCR data for strawberry vein bending virus (SVBV).
